# Complete Chloroplast Genomes and Phylogenetic Relationships of *Bougainvillea spectabilis* and *Bougainvillea glabra* (Nyctaginaceae)

**DOI:** 10.3390/ijms241713044

**Published:** 2023-08-22

**Authors:** Huihui Zhang, Tao Huang, Qi Zhou, Qianqian Sheng, Zunling Zhu

**Affiliations:** 1 College of Landscape Architecture, Nanjing Forestry University, Nanjing 210037, China; zhanghuihui@njfu.edu.cn (H.Z.); bougainvillea97@163.com (T.H.); zhouqi514@njfu.edu.cn (Q.Z.); qqs@njfu.edu.cn (Q.S.); 2 Co-Innovation Center for Sustainable Forestry in Southern China, Nanjing Forestry University, Nanjing 210037, China; 3 Jinpu Research Institute, Nanjing Forestry University, Nanjing 210037, China

**Keywords:** *Bougainvillea*, Nyctaginaceae, chloroplast genome, genome comparative analysis, phylogeny

## Abstract

*Bougainvillea* L. (Nyctaginaceae) is a South American native woody flowering shrub of high ornamental, economic, and medicinal value which is susceptible to cold damage. We sequenced the complete chloroplast (cp) genome of *B. glabra* and *B. spectabilis*, two morphologically similar *Bougainvillea* species differing in cold resistance. Both genomes showed a typical quadripartite structure consisting of one large single-copy region, one small single-copy region, and two inverted repeat regions. The cp genome size of *B. glabra* and *B. spectabilis* was 154,520 and 154,542 bp, respectively, with 131 genes, including 86 protein-coding, 37 transfer RNA, and 8 ribosomal RNA genes. In addition, the genomes contained 270 and 271 simple sequence repeats, respectively, with mononucleotide repeats being the most abundant. Eight highly variable sites (*psbN*, *psbJ*, *rpoA*, *rpl22*, *psaI*, *trnG-UCC*, *ndhF*, and *ycf1*) with high nucleotide diversity were identified as potential molecular markers. Phylogenetic analysis revealed a close relationship between *B. glabra* and *B. spectabilis*. These findings not only contribute to understanding the mechanism by which the cp genome responds to low-temperature stress in *Bougainvillea* and elucidating the evolutionary characteristics and phylogenetic relationships among *Bougainvillea* species, but also provide important evidence for the accurate identification and breeding of superior cold-tolerant *Bougainvillea* cultivars.

## 1. Introduction

Species of the genus *Bougainvillea* L., belonging to the family Nyctaginaceae, are of high horticultural ornamental value. They are tropical and subtropical woody vines characterized by vibrant bracts, a long flowering period, and high stress tolerance, making them ideal ornamental horticultural plants [1]. Recent studies have discovered that *Bougainvillea* potentially has anti-inflammatory, anticancer, antioxidant, antimicrobial, and antihyperglycemic properties [2,3,4,5,6]. This plant group has attracted widespread attention in horticulture, the pharmaceutical industry, and environmental research [7]. *Bougainvillea* is native to Peru, southern Argentina, and Brazil in South America, but is widely cultivated as landscape plants in other warm climate regions such as the Pacific Islands, Southeast Asia, the Mediterranean, Australia, and the Caribbean Islands [8]. The genus comprises approximately 18 species, among which *Bougainvillea spectabilis* Willdenow, *Bougainvillea glabra* Choisy, and *Bougainvillea peruviana* Humboldt and Bonpland are native species and serve as breeding materials for major horticultural cultivars [9]. By hybridizing and mutating these three native species and one hybrid species, *Bougainvillea* x *buttiana* Holttum & Standley, many modern horticultural cultivars with different colors, shapes, and bract sizes have been developed. Currently, there are more than 400 *Bougainvillea* cultivars worldwide. However, the frequent hybridization of *Bougainvillea* species because of commercial demands has led to intricate genetic relationships among many hybrid varieties, resulting in unclear phylogenetic and evolutionary relationships [1,10]. Additionally, due to their origin in South America, *Bougainvillea* plants exhibit a relatively low tolerance to cold temperatures. They are susceptible to cold-related damage throughout their growth, significantly constraining their potential for promotion, application, and the overall development of the *Bougainvillea* industry chain.

Chloroplasts (cps), which are the organelles responsible for photosynthesis in most green plants, participate in developmental processes and secondary metabolic activities, and coordinate gene expression between organelles and the nuclear genome [11,12]. More and more studies have shown that cps play a very important role in plants’ resistance to various environmental stresses [13]. They act as sensors of environmental stresses, connecting diverse stress responses and cellular signaling pathways [14]. The cp genome of angiosperms typically exhibits a characteristic quadripartite structure, consisting of a large single-copy region (LSC), a small single-copy region (SSC), and a pair of inverted repeat regions (IRa and IRb) [15]. It is characterized by structural stability, high conservation, slow molecular evolution, low molecular weight, and maternal inheritance, making it widely applicable in research areas such as molecular marker development and phylogenetics [13,16,17]. Remarkably, research reports regarding the genome of *Bougainvillea* remain conspicuously absent, which seriously hinders the process of improving its cold resistance. However, the cp genome of *Bougainvillea* is relatively small and simple. Therefore, studying the cp genome of *Bougainvillea* can help promote the improvement of its cold resistance, especially in terms of molecular marker selection. Although there have been studies on the cp genomes of species within this genus [7,18,19,20], current research has predominantly focused on *Bougainvillea* species, emphasizing the study of the phylogenetic and classification of *Bougainvillea* from the perspective of plastids. Research reports on the cp genome structure, composition, and variation characteristics among key cultivated varieties are very limited. Furthermore, the response of cps to low-temperature stress in *Bougainvillea* remains to be unveiled.

*Bougainvillea glabra* and *B. spectabilis* are well-known cultivated *Bougainvillea* varieties [21]. Strong evidence indicates that the morphological and horticultural diversity of *Bougainvillea* is primarily due to the variability within *B. glabra* and *B. spectabilis*, which have undergone indistinguishable hybridization with each other and with *B. peruviana*. *Bougainvillea glabra* and *B. spectabilis* are highly similar in terms of their basic morphological characteristics, which has consequently led to confusion in the identification of their cultivated varieties. The former has elliptical, hairless leaves and the flower bracts are covered with fine pubescence or are smooth, whereas the latter has larger, ovate leaves with hairs and the flower bracts are covered with long stiff or soft hairs [22,23]. It was not until the mid-1980s that botanists classified them as distinct species [24]. Studies have shown that *B. glabra* exhibits the strongest cold resistance and has the widest range of applications, whereas *B. spectabilis* has the poorest cold resistance [25,26]. ‘Brasiliensis’ and ‘Auratus’ are excellent cultivars of *B. glabra* and *B. spectabilis*, respectively.

In this study, we sequenced the complete cp genomes of *B. glabra* ‘Brasiliensis’ and *B. spectabilis* ‘Auratus’, two morphologically similar *Bougainvillea* cultivars differing in cold resistance. We comparatively analyzed the cp genomes of *B. glabra*, *B. spectabilis*, and other *Bougainvillea* species aiming to explore the differences in cp genome structure, composition, and variation characteristics. We also studied the phylogenetic relationships among these two and other *Bougainvillea* species. The results of this study not only establish a foundation for understanding the mechanism by which the cp genome responds to low-temperature stress in *Bougainvillea*, but also provide important evidence for the accurate identification and breeding of superior cold-tolerant *Bougainvillea* cultivars.

## 2. Results

### 2.1. Basic Characteristics and Comparative Analysis of the cp Genomes

**Figure 1:**
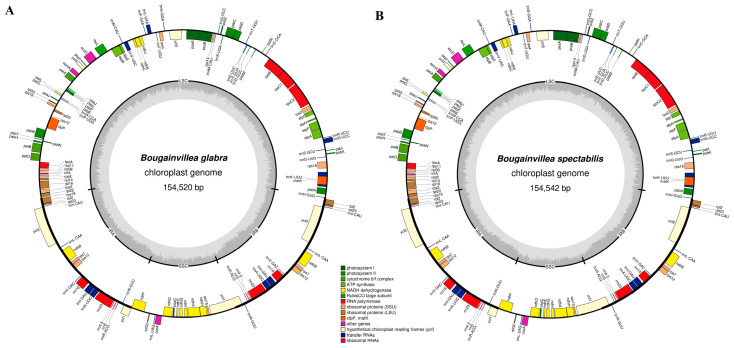
Genome maps of the (**A**) *B. glabra* and (**B**) *B. spectabilis* cp genomes. Genes placed outside the circle are transcribed clockwise, whereas genes inside the circle are transcribed counterclockwise. Gene colors differentiate protein-coding genes based on their respective functions. LSC, large single-copy region; SSC, small single-copy region; IRA and IRB, two inverted repeats; GC content, dark grey area in inner circle; AT content, light grey area in inner circle.

The cp genomes of *B. glabra* and *B. spectabilis* were 154,520 and 154,542 base pairs (bp) in length, respectively (Figure 1). These cp genomes, similar to those of other *Bougainvillea* species, were covalently closed double-stranded circular molecules with a typical quadripartite structure comprising (i) an LSC with a length of 85,688 and 85,695 bp, respectively, accounting for 55.5% of the total genome length in both species; (ii) an SSC with a length of 18,078 and 18,077 bp, respectively, accounting for 11.7% of the total genome length in both species; and (iii) a pair of IRs separating the SSC and LSC regions, with a size of 25,377 and 25,385 bp, respectively, covering 16.4% of the total genome in both species.

**Figure 2:**
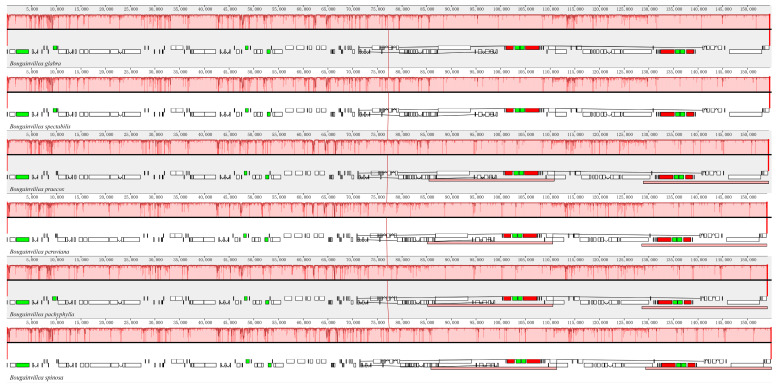
Co-linearity analysis of cp genomes among six *Bougainvillea* species. Within the alignments, local collinear blocks are represented by blocks of the same color connected by lines.

A comparative analysis of the cp genomes of *B. glabra* and *B. spectabilis* and four related *Bougainvillea* species revealed that the cp genome size ranged from 153,966 (*B. peruviana*) to 154,872 bp (*B. spinosa*) (Table 1). Their gene structure, GC content, gene number, mRNA, tRNA, and rRNA were similar, indicating a slow evolution of species within *Bougainvillea*. The GC content of the cp genomes of *B. glabra* and *B. spectabilis* was identical (36.46%). Co-linearity analysis using Mauve software (http://darlinglab.org/mauve, accessed on 24 March 2023) revealed that the structure and gene arrangement sequences of the cp genomes among the six species of *Bougainvillea* were largely similar, with no evident gene rearrangements or inversions. This indicated a high conservation of cp genome sequences in *Bougainvillea* species (Figure 2).

### 2.2. Gene Composition of the cp Genomes

Gene annotation revealed that both the *B. glabra* and *B. spectabilis* cp genomes contained 131 genes, including 86 protein-coding, 37 transfer (t)RNA, and 8 ribosomal (r)RNA genes (Table 1 and Table 2). These genes could be categorized into four groups: photosynthesis-related genes, self-expression-related genes, other genes, and unknown genes. The types and number of genes in these four categories were identical between the *B. glabra* and *B. spectabilis* cp genomes. There were 45 photosynthesis-related genes, including five (*ndhA*, *ndhB*, *petB*, *petD*, and *atpF*) with introns. *ndhB* was present in two copies in the IR regions. There were 74 self-expression-related genes, with one intron each in *rpl16*, *rps16*, *rpoC1*, *trnA-UGC*, *trnI-GAU*, *trnK-UUU*, *trnL-UAA*, and *trnV-UAC* and two introns in *rps12*. Fifteen genes (*rpl2*, *rpl23*, *rps7*, *rps12*, *rrn4.5*, *rrn5*, *rrn16*, *rrn23*, *trnA-UGC*, *trnI-CAU*, *trnI-GAU*, *trnL-CAA*, *trnN-GUU*, *trnR-ACG*, and *trnV-GAC*) were present in two copies in the IR regions. *clpP* in the ‘other genes’ category contained two introns. The unknown genes *ycf1* and *ycf2* were located in the IR regions and existed in two copies, whereas *ycf3* contained two introns (Figure 1, Table 2, Tables S1 and S2). Except for three introns with different lengths, the remaining introns had the same length in both species. *ndhB* had two introns of the same length in each species: 660 bp in *B. glabra* and 668 bp in *B. spectabilis*. Additionally, the introns in *rps16* and *petB* of *B. glabra* were 887 and 777 bp in size, respectively, which were one base pair longer than those in *B. spectabilis* (Tables S1 and S2).

### 2.3. Codon Usage

**Figure 3:**
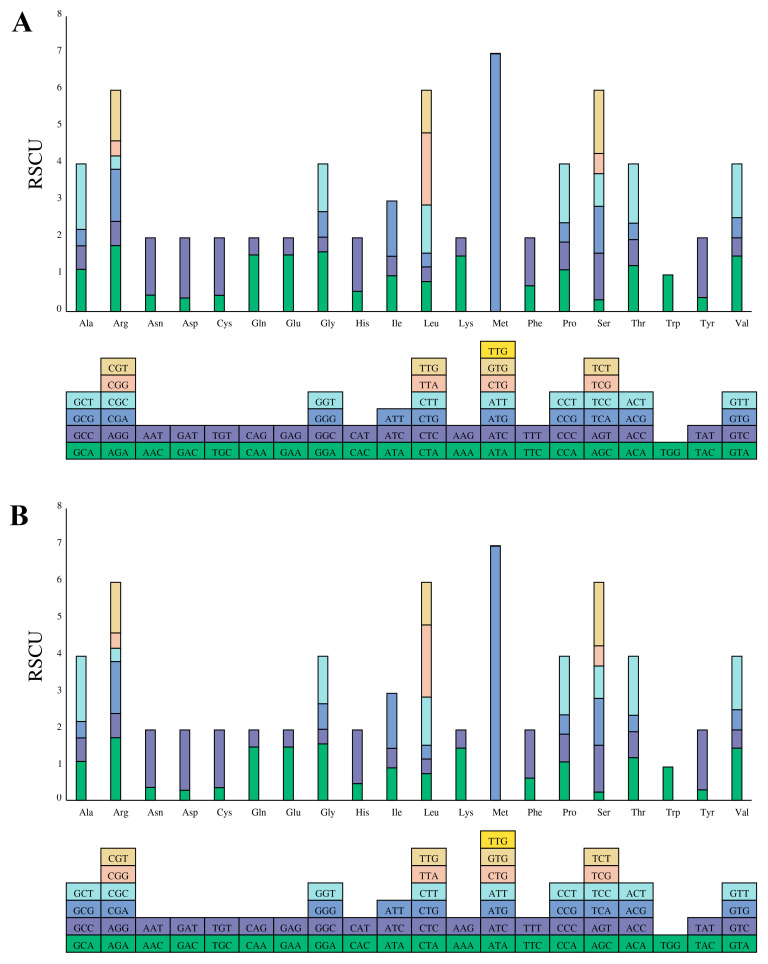
Relative synonymous codon usage (RSCU) values of 20 amino acids and stop codons in the protein-coding genes of the (**A**) *B. glabra* and (**B**) *B. spectabilis* cp genomes. Boxes below the graphs represent all codons encoding each amino acid. The colors of the histograms correspond to the colors of the codons.

Relative synonymous codon usage (RSCU) was used to assess the usage of synonymous codons in the coding sequences, with a higher RSCU value indicating stronger preference [27]. Statistical analysis of the codon numbers and RSCU in the cp DNA of *B. glabra* and *B. spectabilis* revealed that they shared the same number of codons (26,599) and different amino acid types encoded by these codons (21). With respect to the codon numbers encoding other amino acid types, the codon numbers were the same except for lysine (Lys) and asparagine (Asn), which had different codon numbers (1477 and 1296 in *B. glabra*, 1476 and 1297 in *B. spectabilis*, respectively). Leucine (Leu) was the most abundant amino acid, with 2800 codons (accounting for 10.53% of the total codons), followed by isoleucine (Ile) with 2317 codons (8.71% of the total). Cysteine (Cys) was the least abundant, with 297 codons (1.12% of the total) (Table S3). As shown in Figure 3, except for tryptophan (Trp), all amino acids were encoded by two or more synonymous codons, and methionine (Met) was encoded by seven synonymous codons. The preferred synonymous codons (RSCU > 1) mainly ended with A or U, i.e., A/T bases.

### 2.4. Simple Sequence Repeat Analysis

**Figure 4:**
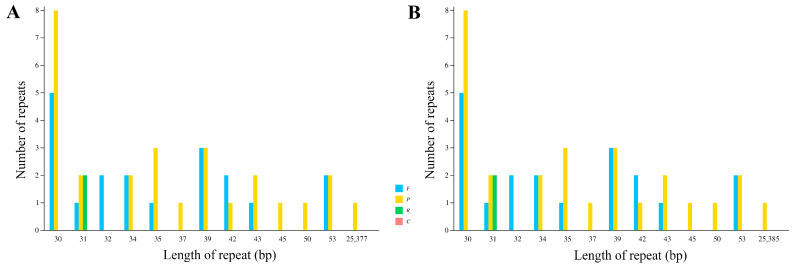
Numbers of different repeats in the cp genomes of (**A**) *B. glabra* and (**B**) *B. spectabilis*. F, forward repeat; P, palindromic repeat; R, reverse repeat; C, complement repeat.

Three types of repetitive sequences were detected in the cp genomes of *B. glabra* and *B. spectabilis*: forward repeats, reverse repeats, and palindromic repeats. There were 19 forward repeats, 27 palindromic repeats, and 2 reverse repeats in both species (Figure 4, Tables S4 and S5).

**Figure 5:**
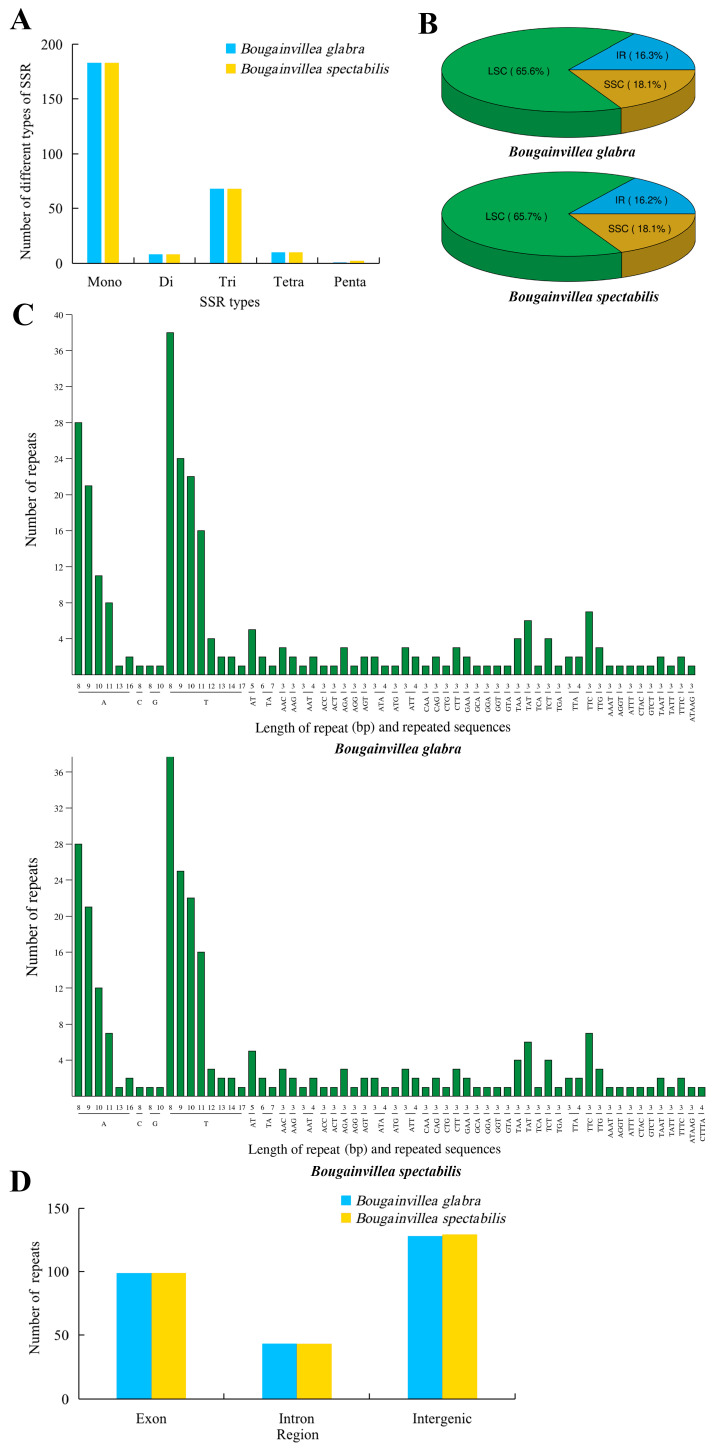
Analysis of simple sequence repeats (SSRs) in the cp genomes of *B. glabra* and *B. spectabilis*. (**A**) Number of different types of SSRs identified in the cp genomes. (**B**) SSR distributions in the LSC, SSC, and IR regions of the cp genomes. (**C**) Frequencies of various SSR types identified in the cp genomes. (**D**) Positional distribution of SSRs in *B. glabra* and *B. spectabilis*.

In total, 270 and 271 simple sequence repeat (SSR) loci were identified in the cp genomes of *B. glabra* and *B. spectabilis*, respectively. Among them, there were 183 mononucleotide repeats (67.78% and 67.53% of the total, respectively), 8 dinucleotide repeats (2.96% and 2.95%), 68 trinucleotide repeats (25.19% and 25.09%), 10 tetranucleotide repeats (3.70% and 3.69%), and 1 and 2 pentanucleotide repeats (0.37% and 0.74%) (Figure 5A, Tables S6 and S7). In the LSC region, there were 177 and 178 SSR loci (65.60% and 65.70%) in *B. glabra* and *B. spectabilis*, respectively, whereas the SSC region had 49 SSR loci (18.10%) and the IR region had 44 SSR loci (16.30%) in both species (Figure 5B). The majority of single-base repeats were A/T, and the dinucleotide repeats were mostly AT/TA (Figure 5C). A total of 128 (47.41%) and 129 (47.60%) SSRs were located in intergenic regions in *B. glabra* and *B. spectabilis*, respectively, whereas 99 SSRs (36.67% and 36.53%) were located in protein-coding genes and 43 SSRs (15.92% and 15.87%) were located in introns (Figure 5D).

### 2.5. Nucleotide Diversity of Genes

**Figure 6:**
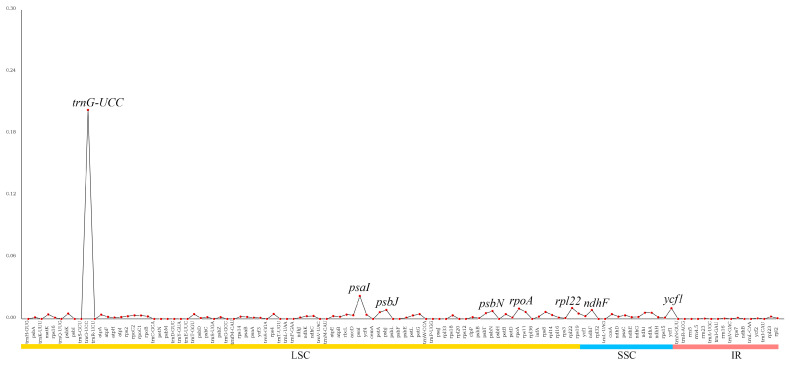
Nucleotide polymorphism analysis of the cp genomes of *B. glabra* and *B. spectabilis*. Names of protein-coding genes and genes of the intergenic region are along the X-axis, and the nucleotide diversity (Pi) value in each window is along the Y-axis.

Nucleotide diversity (Pi) values for *B. glabra* and *B. spectabilis* were calculated using DnaSP software v5.10.1. The results showed that the Pi values in the two cp genomes ranged from 0 to 0.20282, with an average of 0.00401. Eight highly variable regions with Pi > 0.007 were detected. Among them, six were located in the LSC region (*psbN*, *psbJ*, *rpoA*, *rpl22*, *psaI*, and *trnG-UCC*) and two were located in the SSC region (*ndhF* and *ycf1*) (Figure 6).

### 2.6. Analysis of IR Boundary Changes

**Figure 7:**
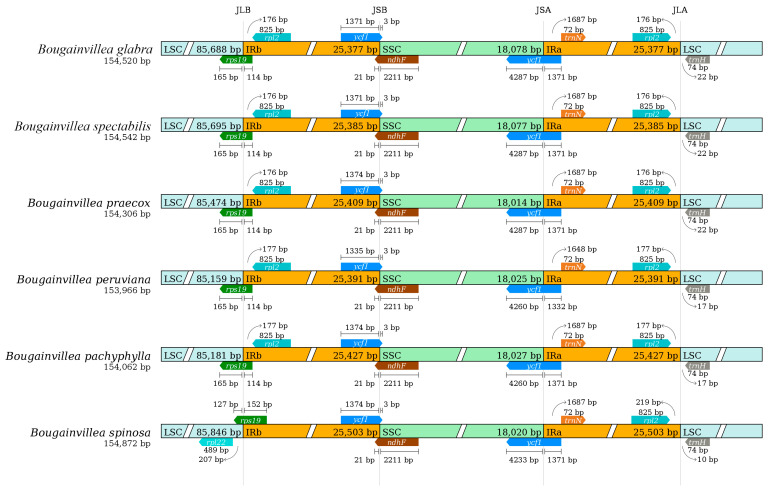
Comparison of IR boundaries in the cp genomes of six *Bougainvillea* species. JLA, junction between LSC and IRa; JLB, junction between LSC and IRb; JSA, junction between SSC and IRa; JSB, junction between SSC and IRb.

As shown in Figure 7, the IR boundaries exhibited a high degree of conservation between *B. glabra* and *B. spectabilis*. The gene content and expansion extent of the boundary regions were identical. When the cp genomes were compared with those of four other *Bougainvillea* species, all six species were found to have the same genes at the boundaries, but with slightly different lengths.

The LSC/IRb (JLB) boundaries of all six species were located in the *ycf1*-coding region. Except for *B. spinosa*, whose *ycf1* gene crossed the JLB boundary by 152 bp, the *ycf1* genes of the other five species crossed the JLB boundary by 114 bp. The expansion range of the SSC/IRb (JSB) boundary showed that in all six *Bougainvillea* species, the JSB boundary was located between *ycf1* and *ndhF*, with slight differences in the extent of expansion. The *ycf1* genes of all six species extended 3 bp beyond the boundary into the SSC region. The IRb region was 1371 bp in length in *B. glabra* and *B. spectabilis*; 1374 bp in *B. praecox*, *B. pachyphylla*, and *B. spinosa*; and 1335 bp in *B. peruviana*. *ndhF* was located near the boundary on the SSC side, and the *ndhF* genes of all species extended 21 bp beyond the boundary into the IRb region. The expansion range of the SSC/IRa (JSA) boundary showed that the JSB boundary in all six *Bougainvillea* species was located within *ycf1*, with *trnN* on the right side, but with slight differences in the extent of expansion. The expansion range of the LSC/IRa (JLA) boundary showed that the JLA boundary had the same genes, with *rpl2* on the left side and *trnH* on the right side, but with slight differences in the extent of expansion. *rpl2* of *B. glabra*, *B. spectabilis*, and *B. praecox* was located 176 bp from the boundary, whereas *trnH* was located 22 bp from the boundary. *rpl2* of *B. peruviana* and *B. pachyphylla* was located 177 bp from the boundary, and *trnH* was located 17 bp from the boundary. *rpl2* of *B. spinosa* was located 219 bp from the boundary, and *trnH* was located 10 bp from the boundary.

### 2.7. Phylogenetic Relationships

**Figure 8:**
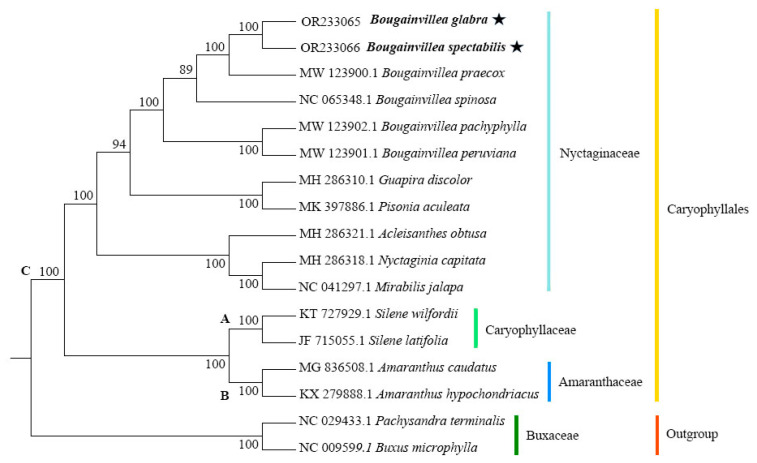
Maximum likelihood phylogenetic tree reconstructed based on the complete cp genome sequences of 17 species. *Bougainvillea microphylla* and *Pachysandra terminalis* were used as the outgroup. Numbers represent bootstrap values (%). A, B and C represent three branches formed by Caryophyllales species. The black stars represent the positions of *B. glabra* and *B. spectabilis*.

To determine the phylogenetic positions and relationships of the cp genomes of *B. glabra* and *B. spectabilis*, the two reassembled *Bougainvillea* cp genomes were compared with the published cp genomes of 15 Caryophyllales species, and a phylogenetic tree was constructed (Figure 8). The results showed high support (>90%) for all branch nodes except one. The outgroup species *Buxus microphylla* and *Pachysandra terminalis* formed one branch, whereas the 15 Caryophyllales (including Caryophyllaceae, Amaranthaceae, and Nyctaginaceae) species formed a larger branch, clearly distinct from the outgroup. The branch formed by the two Caryophyllaceae species *Silene wilfordii* and *Silene latifolia* (branch A) was sister to the branch formed by the Amaranthaceae species *Amaranthus hypochondriacus* and *Amaranthus caudatus* (branch B). The branch consisting of branch A and branch B formed a sister group with the larger branch C formed by 11 other Nyctaginaceae species. Within branch C, *Nyctaginia capitata*, *Mirabilis jalapa*, and *Acleisanthes obtusa* formed a sister group with 100% support. *Guapira discolor* and *Pisonia aculeata* were separated on another branch, and the remaining six *Bougainvillea* species formed a sister group. *Bougainvillea pachyphylla* and *B. peruviana* were basal to the Nyctaginaceae clade. *Bougainvillea glabra* and *B. spectabilis* formed a sister group, which was sister to *B. praecox* with 100% support. The branch formed by *B. glabra*, *B. spectabilis*, and *B. praecox* was sister to *B. spinosa* with 89% support.

## 3. Discussion

For the first time, this study sequenced, assembled, and analyzed the cp genomes of *B. glabra* ‘Brasiliensis’ and *B. spectabilis* ‘Auratus’, two morphologically similar *Bougainvillea* cultivars differing in cold resistance. The results revealed that the cp genomes of these two species possess a typical quadripartite structure, with one LSC, one SSC, and two IR regions, consistent with the cp genome structures of other *Bougainvillea* species and the most common structure in plant cp genomes [7,18,19]. The cp genome size of *B. glabra* and *B. spectabilis* was 154,520 and 154,542 bp, respectively, which was relatively large for *Bougainvillea*. The size of the sequenced cp genomes was found to be similar to the size of the earlier reported cp genomes of *B. glabra* (154,536 bp; 154,763 bp) and *B. spectabilis* (154,541 bp) [18,20]. *Bougainvillea glabra* and *B. spectabilis* exhibit the same total GC content in their cp genomes, and the GC content is significantly higher in the IR region than in the LSC and SSC regions. GC content plays an important role in genome variation, and its uneven distribution may contribute to the conservation of the LSC, SSC, and IR regions. Additionally, *B. glabra* and *B. spectabilis* show consistency in the number of total, protein-coding, rRNA and tRNA genes and introns, indicating a high similarity in their cp genome sequences, which partially explains the similarity in their morphological characteristics.

During biological evolution, codon usage bias is commonly observed among species and can be used to infer phylogenetic relationships among different species or within the same genus [28]. The cp genomes of *B. glabra* and *B. spectabilis* consist of 26,599 codons, with Leu being the most frequently encoded amino acid. The RSCU values showed that the majority of optimal synonymous codons end with A or U, leading to an increased AT content in the genes, supporting the widespread occurrence of A/T codon bias in the cp genomes of higher plants [29]. These results are consistent with those from previous studies on codon usage bias in the cp genomes of *Bougainvillea* species [7,18,19] and suggest that it may be a result of natural selection and gene mutation [30].

SSRs in plant cp genomes are characterized by their abundance, high conservation, and rich genetic information, and variations in their copy numbers can serve as important molecular markers for studying plant polymorphisms, population structure, and population genetic evolution [31]. SSR analysis of the cp genomes of *B. glabra* and *B. spectabilis* revealed 270 and 271 SSR loci, respectively, with mononucleotide repeats, mainly composed of poly-A and poly-T sequences, being the most abundant. This may explain the differences in the base composition of the cp genomes in these two species. Previous studies have also revealed that plastid SSRs are generally composed of poly-A and poly-T repeats and rarely contain guanine (G) and cytosine (C) repeats [32,33]. No hexanucleotide or longer repeats were detected in either species, which is consistent with the findings of Bautista et al. [7], but differs from the results of Yang et al. [19], who detected no tetranucleotide or longer repeats. This difference may be due to different parameter settings in SSR analysis, as Yang et al. [19] set the minimum repeat number for tetranucleotide to hexanucleotide repeats to five. In addition to SSRs, we identified eight highly variable regions in *B. glabra* and *B. spectabilis* using a sliding window approach, six of which were located in the LSC region and the remaining in the SSC region. This indicates that the IR regions of the two genomes are relatively conserved, which is consistent with the research results of Bautista et al. [7]. This is possibly due to the corrective effect of repeated genes in the IR regions on variations [34]. Among these highly variable sites, protein-coding regions *ycf1* and *ndhF* are also highly variable in *Bougainvillea* plants and other plant species, making them recommended candidate regions for DNA barcoding [35,36]. Other fragments, particularly *trnG-UCC*, also exhibit high variability, suggesting their potential as DNA barcoding regions in *Bougainvillea* and suggesting directions for future research.

The contraction and expansion of the IR regions are major factors causing variation in the size of angiosperm cp genomes, as well as gene variation and loss, and pseudogene formation. Therefore, the cp genome size varies among species [37,38,39]. The IR region in angiosperm cp genomes is typically between 20,000 and 30,000 bp, and longer IR regions result in less impact from structural rearrangements on the cp genome [40]. The IR regions of *B. glabra* and *B. spectabilis* were 25,377 and 25,385 bp, respectively, falling within the longer range, indicating higher conservation in this region. Species with minor differences in cp genome junctions are generally closely related [41]. The JLB, JSB, JSA, and JLA boundaries of *B. glabra* and *B. spectabilis* share identical flanking genes, and the expansion lengths of each boundary gene sequence are also consistent. This suggests a high conservation of the IR boundaries between *B. glabra* and *B. spectabilis* and indicates a close phylogenetic relationship. *Bougainvillea glabra*, *B. spectabilis*, and four other *Bougainvillea* species share the same genes at the boundaries, but there are slight differences in the contraction and expansion lengths of the genes, indicating that the contraction and expansion of the IR boundaries in *Bougainvillea* cp genomes are relatively conserved.

To determine the phylogenetic relationship between *B. glabra* and *B. spectabilis* and their systematic positions within *Bougainvillea*, a phylogenetic tree was constructed based on the complete cp genomes of *B. glabra*, *B. spectabilis*, and 15 Caryophyllales species. The results showed that all *Bougainvillea* species formed a major clade, with *B. pachyphylla* and *B. peruviana* as the basal groups of the genus, which is consistent with the findings of Bautista et al. [7] and Bautista et al. [18]. *Bougainvillea glabra* and *B. spectabilis* formed a sister group, indicating a close relationship between them, and this branch was sister to *B. praecox* with 100% support, suggesting a relatively close phylogenetic relationship.

## 4. Materials and Methods

### 4.1. Sampling, DNA Extraction, and Sequencing

*Bougainvillea glabra* ‘Brasiliensis’ (voucher specimen: NJFU220918) and *B. spectabilis* ‘Auratus’ (voucher specimen: NJFU220919) plants were obtained from Zhangzhou Shengxiang Landscape and Greening Co., Ltd. (Zhang’zhou, China) and planted at Nanjing Forestry University (118°81 E, 32°07 N) (Nanjing, China). Voucher specimens were deposited in the VR Laboratory, College of Landscape Architecture, Nanjing Forestry University. Healthy mature leaves were collected from a single plant of both *B. glabra* and *B. spectabilis*, rapidly frozen in liquid nitrogen, and stored at −80 °C until use. Total DNA was extracted from the leaves using a modified cetyltrimethylammonium bromide method [42]. The volume and concentration of *B. glabra* ‘Brasiliensis’ were 40 μL and 54.59 ng/μL, respectively, and those of *B. spectabilis* ‘Auratus’ were 40 μL and 43.86 ng/μL, respectively. After a successful quality assessment of the DNA, it was mechanically disrupted using an ultrasonic homogenizer. Fragment purification, end repair, A-tailing of the 3′ ends, and adapter ligation were performed to generate sequencing libraries. The libraries were sequenced using the Illumina NovaSeq PE150 platform (Genepioneer Biotechnologies, Nanjing, China).

### 4.2. Chloroplast Genome Assembly and Annotation

We used fastp v0.20.0 (https://github.com/OpenGene/fastp (accessed on 24 March 2023)) software [43] to filter the raw data, with the following filtering criteria: (1) sequencing connectors and primer sequences from reads; (2) reads with an average quality value of less than Q5; and (3) reads with a quantity of N greater than 5. After removing adapters and low-quality data from the raw reads, the cp genomes were assembled using SPAdes software v3.10.1 (http://cab.spbu.ru/software/spades/ (accessed on 24 March 2023)) [44], with k-mer sizes of 55, 87, and 121. Contigs were scaffolded using SSPACE v2.0 (https://www.baseclear.com/services/bioinformatics/basetools/sspace-standard/ (accessed on 24 March 2023)) [45]. The software Gapfiller v2.1.1 (https://jaist.dl.sourceforge.net/project/gapfiller/v2.1.1/gapfiller-2.1.1.tar.gz (accessed on 24 March 2023)) was used to fill gaps [46].

Two methods were used for cp genome annotation. First, the cp coding sequences were annotated using Prodigal v2.6.3 (https://www.github.com/hyattpd/Prodigal (accessed on 24 March 2023)), rRNAs were predicted using HMMER software v3.1b2 (http://www.hmmer.org/ (accessed on 24 March 2023)) [47], and tRNAs were predicted using ARAGORN v1.2.38 (http://130.235.244.92/ARAGORN/ (accessed on 24 March 2023)) [48]. Second, gene sequences from closely related species available in the National Center for Biotechnology Information (NCBI) database were extracted and compared with the assembled sequences using BLAST v2.6 (https://blast.ncbi.nlm.nih.gov/Blast.cgi (accessed on 24 March 2023)) to obtain an alternative annotation [49]. The two annotation results were manually inspected to identify discordant gene annotations, remove erroneous and redundant annotations, and determine the boundaries of multi-exonic genes, resulting in the final annotation. The cp genome maps were generated using OGDraw (https://chlorobox.mpimp-golm.mpg.de/OGDraw.html (accessed on 24 March 2023)) [50]. The cp genome sequences have been deposited in GenBank under accession numbers OR233065 (*B. glabra*) and OR233066 (*B. spectabilis*). Mauve software (http://darlinglab.org/mauve, accessed on 24 March 2023) [51] was used for a global comparison of six *Bougainvillea* species and gene rearrangement in the genomes was detected using collinearity analysis. Codon usage was analyzed using CodonW (http://codonw.sourceforge.net/, accessed on 24 March 2023) [52].

### 4.3. Analysis of SSRs, Nucleotide Polymorphisms, and IR Boundary Changes

Vmatch software v2.3.0 (http://www.vmatch.de/ (accessed on 24 March 2023)), in combination with Perl scripts, was used to identify types of repetitive sequences (forward, palindromic, reverse, complement). The parameter settings were as follows: minimum length = 30 bp and Hamming distance = 3 [53]. MISA v1.0 (MIcroSAtellite identification tool, http://pgrc.ipk-gatersleben.de/misa/misa.html (accessed on 24 March 2023)) was used to identify the types and numbers of SSR loci in the cp genomes [54]. The parameter settings were as follows: mononucleotides ≥ 8; dinucleotides ≥ 5; and trinucleotides, tetranucleotides, pentanucleotides, and hexanucleotides ≥ 3. Nucleic acid variations among the cp genomes were determined using DnaSP v5.10 [55]. Differences in boundary sequences were visualized using IRscope (https://ir-scope.shinyapps.io/irapp/ (accessed on 24 March 2023)) [50].

### 4.4. Phylogenetic Analysis

Fifteen published cp genome sequences of Caryophyllales species were downloaded from the NCBI GenBank. *Bougainvillea microphylla* and *P. terminalis* were chosen as outgroups. Multiple sequence alignment was performed using MAFFT v7.427 (auto mode) [56]. Trimmed alignments were obtained using trimAl v1.4.rev15. The maximum likelihood phylogenetic tree was constructed using RAxML v8.2.10 (https://cme.h-its.org/exelixis/software.html (accessed on 24 March 2023)), with the GTRGAMMA model and rapid bootstrap analysis (bootstrap = 1000).

## 5. Conclusions

In this study, the complete cp genomes of ‘Brasiliensis’ and ‘Auratus’, cultivars of *B. glabra* and *B. spectabilis*, respectively, which are important horticultural species, were sequenced and analyzed. The results indicated that the cp genomes of these two species were highly conserved in terms of structure and gene content. A total of 270 and 271 SSR loci were identified in the cp genomes of *B. glabra* and *B. spectabilis*, respectively, alongside eight highly variable regions (*psbN*, *psbJ*, *rpoA*, *rpl22*, *psaI*, *trnG-UCC*, *ndhF*, and *ycf1*), which can serve as potential molecular markers. Phylogenetic analysis showed a close relationship between *B. glabra* and *B. spectabilis*. The findings of this study not only provide important evidence for the further genetic improvement and breeding of cold tolerance in *Bougainvillea* plants and the selection of superior varieties, but also contribute to elucidating the evolutionary and systematic relationships among species in *Bougainvillea*.

## Figures and Tables

**Table 1:** Complete cp genome features of *B. glabra*, *B. spectabilis*, *B. peruviana*, *B. pachyphylla*, *B. praecox*, and *B. spinosa*.

Genome Feature	*Bougainvillea glabra*	*Bougainvillea spectabilis*	*Bougainvillea peruviana*	*Bougainvillea pachyphylla*	*Bougainvillea praecox*	*Bougainvillea spinosa*
Genome size (bp)	154,520	154,542	153,966	154,062	154,306	154,872
LSC length (bp)	85,688	85,695	85,159	85,181	85,474	85,846
SSC length (bp)	18,078	18,077	18,025	18,027	18,014	18,020
IR length (bp)	25,377	25,385	25,391	25,427	25,409	25,503
Number of genes	131	131	131	131	131	131
Number of protein-coding genes	86	86	86	86	86	86
Number of rRNA genes	8	8	8	8	8	8
Number of tRNA genes	37	37	37	37	37	37
GC content (%)	36.5	36.5	36.6	36.5	36.5	36.4
GC content in LSC (%)	34.2	34.2	34.3	34.3	34.3	34.1
GC content in SSC (%)	29.5	29.5	29.6	29.6	29.5	29.4
GC content in IR (%)	42.8	42.8	42.8	42.8	42.8	42.9

LSC, large single copy; SSC, small single copy; IR, inverted repeat.

**Table 2:** Annotated genes and their classification in the cp genomes of *B. glabra* and *B. spectabilis*.

Category	Group	Genes
Photosynthesis	Subunits of photosystem I	*psaA*, *psaB*, *psaC*, *psaI*, *psaJ*
	Subunits of photosystem II	*psbA*, *psbB*, *psbC*, *psbD*, *psbE*, *psbF*, *psbH*, *psbI*, *psbJ*, *psbK*, *psbL*, *psbM*, *psbN*, *psbT*, *psbZ*
	Subunits of NADH dehydrogenase	ndhA *, ndhB * (2), *ndhC*, *ndhD*, *ndhE*, *ndhF*, *ndhG*, *ndhH*, *ndhI*, *ndhJ*, *ndhK*
	Subunits of cytochrome b/f complex	*petA*, petB *, petD *, *petG*, *petL*, *petN*
	Subunits of ATP synthase	*atpA*, *atpB*, *atpE*, atpF *, *atpH*, *atpI*
	Large subunit of rubisco	*rbcL*
	Subunits protochlorophyllide reductase	*-*
Self-replication	Proteins of large ribosomal subunit	*rpl14*, rpl16 *, rpl2 * (2), *rpl20*, *rpl22*, *rpl23* (2), *rpl32*, *rpl33*, *rpl36*
	Proteins of small ribosomal subunit	*rps11*, rps12 ** (2), *rps14*, *rps15*, rps16 *, *rps18*, *rps19*, *rps2*, *rps3*, *rps4*, *rps7* (2), *rps8*
	Subunits of RNA polymerase	*rpoA*, *rpoB*, rpoC1 *, *rpoC2*
	Ribosomal RNAs	*rrn16* (2), *rrn23* (2), *rrn4.5* (2), *rrn5* (2)
	Transfer RNAs	trnA-UGC * (2), *trnC-GCA*, *trnD-GUC*, *trnE-UUC*, *trnF-GAA*, *trnG-GCC*, trnG-UCC *, *trnH-GUG*, *trnI-CAU* (2), trnI-GAU * (2), trnK-UUU *, *trnL-CAA* (2), trnL-UAA *, *trnL-UAG*, *trnM-CAU*, *trnN-GUU* (2), *trnP-UGG*, *trnQ-UUG*, *trnR-ACG* (2), *trnR-UCU*, *trnS-GCU*, *trnS-GGA*, *trnS-UGA*, *trnT-GGU*, *trnT-UGU*, *trnV-GAC* (2), trnV-UAC *, *trnW-CCA*, *trnY-GUA*, *trnfM-CAU*
Other genes	Maturase	*matK*
	Protease	clpP **
	Envelope membrane protein	*cemA*
	Acetyl-CoA carboxylase	*accD*
	c-type cytochrome synthesis gene	*ccsA*
	Translation initiation factor	*infA*
	Other	*-*
Genes of unknown function	Conserved hypothetical chloroplast open reading frame	*ycf1* (2), *ycf2* (2), ycf3 **, *ycf4*

Gene *, gene with one intron; Gene **, gene with two introns; Gene (2), number of copies of multi-copy genes.

## Data Availability

The assembled chloroplast genome sequences of *Bougainvillea glabra* and *Bougainvillea spectabilis* have been uploaded to and deposited in GenBank under accession number OR233065 and OR233066, respectively.

## Supplementary Materials

The supporting information can be downloaded at: https://www.mdpi.com/article/10.3390/ijms241713044/s1.
