# Supplementary material for: Complete Chloroplast Genomes and Phylogenetic Relationships of Bougainvillea spectabilis and Bougainvillea glabra (Nyctaginaceae)
Source: Int J Mol Sci. 2023 Aug 22;24(17):13044. doi: 10.3390/ijms241713044 (PMC10487864; doi:10.3390/ijms241713044)
Supplement: Supplementary file 1 [file ijms-24-13044-s001.zip › ijms-2544166-SI.pdf]

Table S1. Lengths of exons and introns in genes with introns in the *Bougainvillea glabra* chloroplast genome.

Gene	Location	Exon I (bp)	Intron I (bp)	Exon II (bp)	Intron II (bp)	Exon III (bp)
trnK-UUU	LSC	37	2508	35		
rps16	LSC	41	887	226		
trnG-UCC	LSC	23	705	48		
atpF	LSC	145	768	410		
rpoC1	LSC	432	813	1602		
ycf3	LSC	126	766	228	799	153
trnL-UAA	LSC	37	539	50		
trnV-UAC	LSC	39	605	35		
rps12	IRa	114	-	232	543	26
clpP	LSC	71	631	294	776	226
petB	LSC	6	777	642		
petD	LSC	8	722	475		
rpl16	LSC	9	929	399		
ndhB	IRb	777	660	756		

LLC, large single copy; SSC, small single copy; IRs, inverted repeats.

Table S2. Lengths of exons and introns in genes with introns in the *Bougainvillea spectabilis* chloroplast genome.

Gene	Location	Exon I (bp)	Intron I (bp)	Exon II (bp)	Intron II (bp)	Exon III (bp)
trnK-UUU	LSC	37	2508	35		
rps16	LSC	41	886	226		
trnG-UCC	LSC	23	705	48		
atpF	LSC	145	768	410		
rpoC1	LSC	432	813	1602		
ycf3	LSC	126	766	228	799	153
trnL-UAA	LSC	37	539	50		
trnV-UAC	LSC	39	605	35		
rps12	IRa	114	-	232	543	26
clpP	LSC	71	631	294	776	226
petB	LSC	6	776	642		
petD	LSC	8	722	475		
rpl16	LSC	9	929	399		
ndhB	IRb	777	668	756		

Table S3. Coding capacity of protein coding genes (PCGs) and relative synonymous codon usage (RSCU).

AminoAcid	Symbol	Codon	*Bougainvillea glabra*		*Bougainvillea spectabilis*	
			No.	RSCU	No.	RSCU
*	Ter	UAA	50	1.7442	50	1.7442
*	Ter	UAG	21	0.7326	21	0.7326
*	Ter	UGA	15	0.5232	15	0.5232
A	Ala	GCA	398	1.146	398	1.146
A	Ala	GCC	222	0.6392	222	0.6392
A	Ala	GCG	154	0.4436	154	0.4436
A	Ala	GCU	615	1.7712	615	1.7712
C	Cys	UGC	65	0.4378	65	0.4378
C	Cys	UGU	232	1.5622	232	1.5622
D	Asp	GAC	195	0.3658	195	0.3658
D	Asp	GAU	871	1.6342	871	1.6342
E	Glu	GAA	1087	1.5408	1087	1.5408
E	Glu	GAG	324	0.4592	324	0.4592
F	Phe	UUC	543	0.697	543	0.697
F	Phe	UUU	1015	1.303	1015	1.303
G	Gly	GGA	723	1.6212	723	1.6212
G	Gly	GGC	177	0.3968	177	0.3968
G	Gly	GGG	310	0.6952	310	0.6952
G	Gly	GGU	574	1.2868	574	1.2868
H	His	CAC	171	0.549	171	0.549
H	His	CAU	452	1.451	452	1.451
I	Ile	AUA	751	0.9723	751	0.9723
I	Ile	AUC	410	0.531	410	0.531
I	Ile	AUU	1156	1.4967	1156	1.4967
K	Lys	AAA	1115	1.5098	1115	1.5108
K	Lys	AAG	362	0.4902	361	0.4892
L	Leu	CUA	382	0.8184	382	0.8184
L	Leu	CUC	186	0.3984	186	0.3984
L	Leu	CUG	172	0.3684	172	0.3684
L	Leu	CUU	608	1.3026	608	1.3026
L	Leu	UUA	915	1.9608	915	1.9608
L	Leu	UUG	537	1.1508	537	1.1508
M	Met	AUA	0	0	0	0
M	Met	AUC	0	0	0	0
M	Met	AUG	632	6.9888	632	6.9888
M	Met	AUU	0	0	0	0

M	Met	CUG	0	0	0	0
M	Met	GUG	1	0.0112	1	0.0112
M	Met	UUG	0	0	0	0
N	Asn	AAC	289	0.446	289	0.4456
N	Asn	AAU	1007	1.554	1008	1.5544
P	Pro	CCA	307	1.138	307	1.138
P	Pro	CCC	202	0.7488	202	0.7488
P	Pro	CCG	142	0.5264	142	0.5264
P	Pro	CCU	428	1.5868	428	1.5868
Q	Gln	CAA	728	1.5374	728	1.5374
Q	Gln	CAG	219	0.4626	219	0.4626
R	Arg	AGA	473	1.7928	473	1.7928
R	Arg	AGG	172	0.6522	172	0.6522
R	Arg	CGA	373	1.4136	373	1.4136
R	Arg	CGC	95	0.36	95	0.36
R	Arg	CGG	109	0.4134	109	0.4134
R	Arg	CGU	361	1.368	361	1.368
S	Ser	AGC	109	0.3228	109	0.3228
S	Ser	AGU	427	1.2654	427	1.2654
S	Ser	UCA	427	1.2654	427	1.2654
S	Ser	UCC	298	0.8832	298	0.8832
S	Ser	UCG	184	0.5454	184	0.5454
S	Ser	UCU	580	1.7184	580	1.7184
T	Thr	ACA	421	1.2484	421	1.2484
T	Thr	ACC	238	0.7056	238	0.7056
T	Thr	ACG	149	0.442	149	0.442
T	Thr	ACU	541	1.604	541	1.604
V	Val	GUA	526	1.5116	526	1.5116
V	Val	GUC	170	0.4884	170	0.4884
V	Val	GUG	191	0.5488	191	0.5488
V	Val	GUU	505	1.4512	505	1.4512
W	Trp	UGG	468	1	468	1
Y	Tyr	UAC	195	0.3828	195	0.3828
Y	Tyr	UAU	824	1.6172	824	1.6172

RSCU, relative synonymous codon usage.

Table S4. Distribution and location of tandem repeats in the *Bougainvillea glabra* chloroplast genome.

ID	Repeat I Start	Repeat II Start	Type	Size(bp)	E-Value	Gene	Region
1	85689	129144	P	25377	0.00E+00	-	ir
2	92025	92043	F	53	5.24E-17	ycf2;ycf2	IRb;IRb
3	92025	148114	P	53	5.24E-17	ycf2;ycf2	IRb;IRa
4	92043	148132	P	53	5.24E-17	ycf2;ycf2	IRb;IRa
5	148114	148132	F	53	5.24E-17	ycf2;ycf2	IRa;IRa
6	118152	118152	P	50	5.30E-21	ndhD;ndhD	SSC;SSC
7	53723	53723	P	45	7.32E-16	IGS	LSC;LSC
8	108380	108380	P	43	2.89E-11	IGS	IRb;IRb
9	108380	131787	F	43	2.89E-11	IGS	IRb;IRa
10	131787	131787	P	43	2.89E-11	IGS	IRa;IRa
11	99312	121479	F	42	4.37E-14	IGS;ndhA	IRb;SSC
12	121479	140856	P	42	4.37E-14	ndhA;IGS	SSC;IRa
13	44344	121478	F	42	1.08E-10	ycf3;ndhA	LSC;SSC
14	44347	99314	F	39	5.48E-09	ycf3;IGS	LSC;IRb
15	44347	140857	P	39	5.48E-09	ycf3;IGS	LSC;IRa
16	92017	92053	F	39	5.48E-09	ycf2;ycf2	IRb;IRb
17	92017	148118	P	39	5.48E-09	ycf2;ycf2	IRb;IRa
18	92053	148154	P	39	5.48E-09	ycf2;ycf2	IRb;IRa
19	148118	148154	F	39	5.48E-09	ycf2;ycf2	IRa;IRa
20	47168	47168	P	37	3.95E-11	IGS	LSC;LSC
21	8708	8708	P	35	5.97E-10	IGS	LSC;LSC
22	92043	92061	F	35	5.97E-10	ycf2;ycf2	IRb;IRb
23	92043	148114	P	35	5.97E-10	ycf2;ycf2	IRb;IRa
24	92061	148132	P	35	5.97E-10	ycf2;ycf2	IRb;IRa
25	108047	108079	F	34	3.68E-06	IGS	IRb;IRb
26	108047	132097	P	34	3.68E-06	IGS	IRb;IRa
27	108079	132129	P	34	3.68E-06	IGS	IRb;IRa
28	132097	132129	F	34	3.68E-06	IGS	IRa;IRa
29	8337	35891	F	32	4.88E-05	trnS-GCU;trnS-UGA	LSC;LSC
30	39173	41397	F	32	4.88E-05	psaB;psaA	LSC;LSC
31	43368	99336	F	31	1.77E-04	ycf3;IGS	LSC;IRb
32	43368	140843	P	31	1.77E-04	ycf3;IGS	LSC;IRa
33	73104	73104	P	31	1.77E-04	clpP;clpP	LSC;LSC
34	49531	49535	R	31	1.77E-04	IGS	LSC;LSC
35	69320	69322	R	31	1.77E-04	IGS	LSC;LSC
36	8339	46095	P	30	5.82E-09	trnS-GCU;t	LSC;LSC

						rnS-GGA	
37	89078	113939	F	30	5.82E-09	ycf2;IGS	IRb;SSC
38	113939	151102	P	30	5.82E-09	IGS;ycf2	SSC;IRa
39	94713	94747	P	30	5.24E-07	IGS	IRb;IRb
40	94713	145433	F	30	5.24E-07	IGS	IRb;IRa
41	94747	145467	F	30	5.24E-07	IGS	IRb;IRa
42	145433	145467	P	30	5.24E-07	IGS	IRa;IRa
43	99326	121493	F	30	2.28E-05	IGS;ndhA	IRb;SSC
44	121493	140854	P	30	2.28E-05	ndhA;IGS	SSC;IRa
45	10128	36910	F	30	6.38E-04	trnG-UCC;trnG-GCC	LSC;LSC
46	35893	46095	P	30	6.38E-04	trnS-UGA;trnS-GGA	LSC;LSC
47	46207	70640	P	30	6.38E-04	IGS	LSC;LSC
48	66584	66587	P	30	6.38E-04	IGS	LSC;LSC

F, forward repeat; P, palindrome repeat.

Table S5. Distribution and location of tandem repeats in the *Bougainvillea spectabilis* chloroplast genome.

ID	Repeat I Start	Repeat II Start	Type	Size(bp)	E-Value	Gene	Region
1	85696	129158	P	25385	0.00E+00	-	ir
2	92032	92050	F	53	5.24E-17	ycf2;ycf2	IRb;IRb
3	92032	148136	P	53	5.24E-17	ycf2;ycf2	IRb;IRa
4	92050	148154	P	53	5.24E-17	ycf2;ycf2	IRb;IRa
5	148136	148154	F	53	5.24E-17	ycf2;ycf2	IRa;IRa
6	118166	118166	P	50	5.30E-21	ndhD;ndhD	SSC;SSC
7	53731	53731	P	45	7.33E-16	IGS	LSC;LSC
8	108395	108395	P	43	2.89E-11	IGS	IRb;IRb
9	108395	131801	F	43	2.89E-11	IGS	IRb;IRa
10	131801	131801	P	43	2.89E-11	IGS	IRa;IRa
11	99327	121493	F	42	4.38E-14	IGS;ndhA	IRb;SSC
12	121493	140870	P	42	4.38E-14	ndhA;IGS	SSC;IRa
13	44352	121492	F	42	1.08E-10	ycf3;ndhA	LSC;SSC
14	44355	99329	F	39	5.48E-09	ycf3;IGS	LSC;IRb
15	44355	140871	P	39	5.48E-09	ycf3;IGS	LSC;IRa
16	92024	92060	F	39	5.48E-09	ycf2;ycf2	IRb;IRb
17	92024	148140	P	39	5.48E-09	ycf2;ycf2	IRb;IRa
18	92060	148176	P	39	5.48E-09	ycf2;ycf2	IRb;IRa
19	148140	148176	F	39	5.48E-09	ycf2;ycf2	IRa;IRa
20	47176	47176	P	37	3.95E-11	IGS	LSC;LSC
21	8707	8707	P	35	5.97E-10	IGS	LSC;LSC
22	92050	92068	F	35	5.97E-10	ycf2;ycf2	IRb;IRb
23	92050	148136	P	35	5.97E-10	ycf2;ycf2	IRb;IRa
24	92068	148154	P	35	5.97E-10	ycf2;ycf2	IRb;IRa
25	108062	108094	F	34	3.68E-06	IGS	IRb;IRb
26	108062	132111	P	34	3.68E-06	IGS	IRb;IRa
27	108094	132143	P	34	3.68E-06	IGS	IRb;IRa
28	132111	132143	F	34	3.68E-06	IGS	IRa;IRa
29	8336	35899	F	32	4.88E-05	trnS-GCU;trnS-UGA	LSC;LSC
30	39181	41405	F	32	4.88E-05	psaB;psaA	LSC;LSC
31	43376	99351	F	31	1.77E-04	ycf3;IGS	LSC;IRb
32	43376	140857	P	31	1.77E-04	ycf3;IGS	LSC;IRa
33	73112	73112	P	31	1.77E-04	clpP;clpP	LSC;LSC
34	49539	49543	R	31	1.77E-04	IGS	LSC;LSC
35	69328	69330	R	31	1.77E-04	IGS	LSC;LSC
36	8338	46103	P	30	5.83E-09	trnS-GCU;t	LSC;LSC

						rnS-GGA	
37	89085	113954	F	30	5.83E-09	ycf2;IGS	IRb;SSC
38	113954	151124	P	30	5.83E-09	IGS;ycf2	SSC;IRa
39	94720	94754	P	30	5.24E-07	IGS	IRb;IRb
40	94720	145455	F	30	5.24E-07	IGS	IRb;IRa
41	94754	145489	F	30	5.24E-07	IGS	IRb;IRa
42	145455	145489	P	30	5.24E-07	IGS	IRa;IRa
43	99341	121507	F	30	2.28E-05	IGS;ndhA	IRb;SSC
44	121507	140868	P	30	2.28E-05	ndhA;IGS	SSC;IRa
45	10126	36918	F	30	6.39E-04	trnG-UCC;trnG-GCC	LSC;LSC
46	35901	46103	P	30	6.39E-04	trnS-UGA;trnS-GGA	LSC;LSC
47	46215	70648	P	30	6.39E-04	IGS	LSC;LSC
48	66592	66595	P	30	6.39E-04	IGS	LSC;LSC

Table S6. Simple sequence repeats (SSRs) in the *Bougainvillea glabra* chloroplast genome.

ID	Repeat Motif	Length(bp)	Start	End	Region	Gene	ID	Repeat Motif	Length(bp)	Start	End	Region	Gene
1	(A)8	8	131	138	LSC		136	(TTC)3	9	68997	69005	LSC	
2	(TA)7	14	264	277	LSC		137	(TAT)3	9	69346	69354	LSC	
3	(ATA)3	9	431	439	LSC		138	(AAC)3	9	69644	69652	LSC	*rps18*
4	(T)10	10	457	466	LSC		139	(T)8	8	70360	70367	LSC	*rpl20*
5	(A)9	9	1721	1729	LSC		140	(T)13	13	70571	70583	LSC	
6	(T)10	10	1812	1821	LSC		141	(A)16	16	70648	70663	LSC	
7	(A)8	8	3752	3759	LSC	*trnK-UUU*	142	(T)8	8	71884	71891	LSC	*clpP*
8	(T)8	8	3901	3908	LSC	*trnK-UUU*	143	(T)8	8	72044	72051	LSC	*clpP*
9	(A)13	13	4738	4750	LSC		144	(A)9	9	72058	72066	LSC	*clpP*
10	(T)10	10	5819	5828	LSC	*rps16*	145	(A)9	9	72816	72824	LSC	*clpP*
11	(A)10	10	6568	6577	LSC		146	(T)8	8	72938	72945	LSC	*clpP*
12	(T)9	9	6651	6659	LSC		147	(T)11	11	73027	73037	LSC	*clpP*
13	(A)8	8	6680	6687	LSC		148	(T)10	10	73053	73062	LSC	*clpP*
14	(A)9	9	7368	7376	LSC		149	(T)12	12	73103	73114	LSC	*clpP*
15	(T)10	10	7396	7405	LSC		150	(A)10	10	73125	73134	LSC	*clpP*
16	(T)12	12	7407	7418	LSC		151	(A)11	11	73511	73521	LSC	
17	(A)8	8	8041	8048	LSC		152	(A)10	10	73630	73639	LSC	
18	(T)8	8	8253	8260	LSC		153	(TTG)3	9	74706	74714	LSC	*psbB*
19	(A)9	9	8434	8442	LSC		154	(TCT)3	9	75339	75347	LSC	*psbB*
20	(AT)5	10	8596	8605	LSC		155	(T)8	8	75516	75523	LSC	
21	(TAT)3	9	8737	8745	LSC		156	(T)9	9	75588	75596	LSC	

22	(T)12	12	9075	9086	LSC		157	(ATT)4	12	75964	75975	LSC	*psbN*
23	(A)9	9	9191	9199	LSC		158	(TTTC)3	12	76415	76426	LSC	
24	(T)11	11	9480	9490	LSC	*trnG-UCC*	159	(A)10	10	76632	76641	LSC	*petB*
25	(T)9	9	9612	9620	LSC	*trnG-UCC*	160	(T)8	8	76737	76744	LSC	*petB*
26	(T)11	11	9715	9725	LSC	*trnG-UCC*	161	(A)8	8	76933	76940	LSC	*petB*
27	(A)8	8	10270	10277	LSC		162	(A)11	11	76966	76976	LSC	*petB*
28	(GTCT)3	12	11485	11496	LSC	*atpA*	163	(A)11	11	76990	77000	LSC	*petB*
29	(A)10	10	12040	12049	LSC		164	(A)10	10	78108	78117	LSC	
30	(A)9	9	12735	12743	LSC	*atpF*	165	(T)9	9	78368	78376	LSC	*petD*
31	(T)8	8	12784	12791	LSC	*atpF*	166	(T)9	9	78379	78387	LSC	*petD*
32	(AAC)3	9	14913	14921	LSC	*atpI*	167	(T)10	10	79455	79464	LSC	
33	(TTA)4	12	15725	15736	LSC		168	(T)10	10	79734	79743	LSC	*rpoA*
34	(T)9	9	16809	16817	LSC	*rpoC2*	169	(TTC)3	9	79828	79836	LSC	*rpoA*
35	(T)11	11	18699	18709	LSC	*rpoC2*	170	(T)10	10	81670	81679	LSC	
36	(A)8	8	18843	18850	LSC	*rpoC2*	171	(T)11	11	81959	81969	LSC	*rps8*
37	(AT)5	10	20049	20058	LSC	*rpoC2*	172	(T)9	9	82648	82656	LSC	
38	(TTA)3	9	21114	21122	LSC	*rpoC1*	173	(T)8	8	83364	83371	LSC	*rpl16*
39	(TTC)3	9	22305	22313	LSC	*rpoC1*	174	(T)8	8	83834	83841	LSC	*rpl16*
40	(A)8	8	22489	22496	LSC	*rpoC1*	175	(T)8	8	84087	84094	LSC	*rpl16*
41	(T)11	11	22811	22821	LSC	*rpoC1*	176	(A)9	9	84199	84207	LSC	
42	(A)8	8	22967	22974	LSC	*rpoC1*	177	(ATT)3	9	84276	84284	LSC	*rps3*
43	(T)11	11	23188	23198	LSC	*rpoC1*	178	(T)9	9	85780	85788	IR	*rps19*
44	(T)8	8	26328	26335	LSC	*rpoB*	179	(T)11	11	85818	85828	IR	
45	(T)10	10	26432	26441	LSC	*rpoB*	180	(CTT)3	9	85920	85928	IR	*rpl2*

46	(A)8	8	27310	27317	LSC		181	(G)8	8	89118	89125	IR	*ycf2*
47	(TAT)3	9	27398	27406	LSC		182	(A)9	9	90257	90265	IR	*ycf2*
48	(T)10	10	27568	27577	LSC		183	(AGT)3	9	90776	90784	IR	*ycf2*
49	(T)8	8	27787	27794	LSC		184	(TCT)3	9	90913	90921	IR	*ycf2*
50	(A)8	8	27841	27848	LSC		185	(CTT)3	9	91019	91027	IR	*ycf2*
51	(T)10	10	28357	28366	LSC		186	(GGT)3	9	91283	91291	IR	*ycf2*
52	(T)8	8	29310	29317	LSC		187	(TGA)3	9	91519	91527	IR	*ycf2*
53	(A)10	10	29644	29653	LSC		188	(GAA)3	9	92779	92787	IR	*ycf2*
54	(T)9	9	29934	29942	LSC		189	(AGA)3	9	96078	96086	IR	*ndhB*
55	(A)16	16	30136	30151	LSC		190	(AGA)3	9	97497	97505	IR	*ndhB*
56	(T)10	10	30176	30185	LSC		191	(T)8	8	99858	99865	IR	
57	(A)9	9	30500	30508	LSC		192	(AAG)3	9	100408	100416	IR	
58	(T)10	10	30980	30989	LSC		193	(T)11	11	100458	100468	IR	
59	(TTA)3	9	31182	31190	LSC		194	(T)8	8	103328	103335	IR	*trnI-GAU*
60	(TAA)3	9	31242	31250	LSC		195	(T)8	8	104640	104647	IR	*trnA-UGC*
61	(A)8	8	31327	31334	LSC		196	(CTG)3	9	105122	105130	IR	*rrn23*
62	(ATAAG)3	15	32090	32104	LSC		197	(AGGT)3	12	106639	106650	IR	*rrn23*
63	(T)9	9	32269	32277	LSC		198	(CAA)3	9	110183	110191	IR	*ycf1*
64	(ATT)4	12	33047	33058	LSC		199	(T)8	8	110867	110874	IR	*ycf1*
65	(T)9	9	33062	33070	LSC		200	(A)8	8	111752	111759	SSC	*ndhF*
66	(T)9	9	33094	33102	LSC		201	(T)8	8	111869	111876	SSC	*ndhF*
67	(G)10	10	34812	34821	LSC	*psbC*	202	(TAA)3	9	112704	112712	SSC	*ndhF*
68	(T)9	9	35788	35796	LSC		203	(A)8	8	113190	113197	SSC	*ndhF*
69	(ATA)3	9	36814	36822	LSC		204	(A)9	9	113644	113652	SSC	

70	(AT)5	10	36820	36829	LSC		205	(T)8	8	113670	113677	SSC	
71	(TCT)3	9	37652	37660	LSC	*rps14*	206	(T)8	8	114329	114336	SSC	*rpl32*
72	(ATG)3	9	39184	39192	LSC	*psaB*	207	(T)9	9	114491	114499	SSC	
73	(TTG)3	9	40789	40797	LSC	*psaA*	208	(T)11	11	114684	114694	SSC	
74	(GCA)3	9	41082	41090	LSC	*psaA*	209	(T)8	8	114864	114871	SSC	
75	(T)11	11	42411	42421	LSC		210	(T)11	11	114883	114893	SSC	
76	(AT)5	10	42790	42799	LSC		211	(A)8	8	115111	115118	SSC	
77	(T)8	8	42829	42836	LSC		212	(T)8	8	115419	115426	SSC	
78	(T)11	11	42848	42858	LSC		213	(TTC)3	9	116576	116584	SSC	
79	(TATT)3	12	42877	42888	LSC		214	(A)8	8	116630	116637	SSC	*ndhD*
80	(ATT)3	9	42893	42901	LSC		215	(T)8	8	117215	117222	SSC	*ndhD*
81	(A)11	11	42960	42970	LSC		216	(TAT)3	9	117276	117284	SSC	*ndhD*
82	(T)10	10	44014	44023	LSC	*ycf3*	217	(A)9	9	118208	118216	SSC	*ndhD*
83	(T)8	8	44684	44691	LSC	*ycf3*	218	(AAT)4	12	118852	118863	SSC	
84	(A)10	10	45087	45096	LSC	*ycf3*	219	(A)9	9	118864	118872	SSC	
85	(T)9	9	45390	45398	LSC		220	(A)9	9	119396	119404	SSC	
86	(GTA)3	9	45472	45480	LSC		221	(TTC)3	9	121277	121285	SSC	*ndhA*
87	(T)11	11	46213	46223	LSC		222	(T)10	10	121703	121712	SSC	*ndhA*
88	(TA)6	12	47107	47118	LSC		223	(T)8	8	121715	121722	SSC	*ndhA*
89	(A)9	9	47280	47288	LSC		224	(T)8	8	121897	121904	SSC	*ndhA*
90	(T)11	11	47648	47658	LSC		225	(T)9	9	124399	124407	SSC	*rps15*
91	(TA)6	12	47730	47741	LSC		226	(TAA)3	9	124665	124673	SSC	
92	(A)8	8	47799	47806	LSC		227	(ATT)3	9	125145	125153	SSC	*ycf1*
93	(A)9	9	48565	48573	LSC	*trnL-UAA*	228	(T)12	12	125685	125696	SSC	*ycf1*

94	(T)10	10	48632	48641	LSC	*trnL-UAA*	229	(T)8	8	125850	125857	SSC	*ycf1*
95	(T)9	9	50540	50548	LSC		230	(T)11	11	125878	125888	SSC	*ycf1*
96	(AAC)3	9	51197	51205	LSC	*ndhK*	231	(T)10	10	126067	126076	SSC	*ycf1*
97	(T)9	9	51346	51354	LSC		232	(TTA)4	12	126124	126135	SSC	*ycf1*
98	(TAT)3	9	52175	52183	LSC		233	(T)9	9	126403	126411	SSC	*ycf1*
99	(A)8	8	52590	52597	LSC		234	(T)9	9	126496	126504	SSC	*ycf1*
100	(T)8	8	52773	52780	LSC	*trnV-UAC*	235	(TAAT)3	12	126571	126582	SSC	*ycf1*
101	(T)10	10	53698	53707	LSC		236	(T)14	14	126612	126625	SSC	*ycf1*
102	(T)10	10	55692	55701	LSC	*atpB*	237	(T)8	8	127343	127350	SSC	*ycf1*
103	(ATA)4	12	55746	55757	LSC	*atpB*	238	(T)17	17	127541	127557	SSC	*ycf1*
104	(A)8	8	55986	55993	LSC		239	(ATTT)3	12	127580	127591	SSC	*ycf1*
105	(T)8	8	58483	58490	LSC		240	(T)10	10	127679	127688	SSC	*ycf1*
106	(AGT)3	9	59197	59205	LSC	*accD*	241	(T)13	13	127789	127801	SSC	*ycf1*
107	(GGA)3	9	59559	59567	LSC	*accD*	242	(AAT)3	9	128011	128019	SSC	*ycf1*
108	(A)9	9	60238	60246	LSC		243	(T)8	8	128147	128154	SSC	*ycf1*
109	(T)10	10	60511	60520	LSC		244	(T)9	9	128196	128204	SSC	*ycf1*
110	(A)10	10	60548	60557	LSC		245	(A)9	9	128312	128320	SSC	*ycf1*
111	(AAT)4	12	60573	60584	LSC		246	(T)8	8	128676	128683	SSC	*ycf1*
112	(T)8	8	60633	60640	LSC		247	(T)9	9	128959	128967	SSC	*ycf1*
113	(TAAT)3	12	60702	60713	LSC		248	(T)9	9	129089	129097	SSC	*ycf1*
114	(AAAT)3	12	61995	62006	LSC		249	(A)8	8	129335	129342	IR	*ycf1*
115	(T)10	10	62441	62450	LSC		250	(TTG)3	9	130018	130026	IR	*ycf1*
116	(A)9	9	62534	62542	LSC		251	(CTAC)3	12	133557	133568	IR	*rrn23*
117	(A)11	11	62658	62668	LSC	*cemA*	252	(CAG)3	9	135079	135087	IR	*rrn23*

118	(AT)5	10	63702	63711	LSC	*petA*	253	(A)8	8	135562	135569	IR	*trnA-UGC*
119	(CAG)3	9	64258	64266	LSC	*petA*	254	(A)8	8	136874	136881	IR	*trnI-GAU*
120	(A)8	8	64631	64638	LSC		255	(A)11	11	139741	139751	IR	
121	(T)14	14	64697	64710	LSC		256	(CTT)3	9	139793	139801	IR	
122	(A)11	11	65222	65232	LSC		257	(A)8	8	140344	140351	IR	
123	(TAA)3	9	65482	65490	LSC		258	(TTC)3	9	142703	142711	IR	*ndhB*
124	(TTTC)3	12	65845	65856	LSC		259	(TCT)3	9	144123	144131	IR	*ndhB*
125	(A)10	10	66039	66048	LSC	*psbF*	260	(TTC)3	9	147422	147430	IR	*ycf2*
126	(A)8	8	66759	66766	LSC		261	(TCA)3	9	148682	148690	IR	*ycf2*
127	(T)8	8	67457	67464	LSC		262	(ACC)3	9	148918	148926	IR	*ycf2*
128	(AGG)3	9	67642	67650	LSC		263	(AAG)3	9	149182	149190	IR	*ycf2*
129	(A)8	8	67890	67897	LSC		264	(AGA)3	9	149288	149296	IR	*ycf2*
130	(A)10	10	68194	68203	LSC		265	(ACT)3	9	149425	149433	IR	*ycf2*
131	(A)9	9	68252	68260	LSC		266	(T)9	9	149944	149952	IR	*ycf2*
132	(T)8	8	68549	68556	LSC	*psaJ*	267	(C)8	8	151084	151091	IR	*ycf2*
133	(A)8	8	68663	68670	LSC		268	(GAA)3	9	154280	154288	IR	*rpl2*
134	(A)8	8	68765	68772	LSC		269	(A)11	11	154381	154391	IR	
135	(TAT)3	9	68912	68920	LSC		270	(A)9	9	154421	154429	IR	

Table S7. Simple sequence repeats (SSRs) in the *Bougainvillea spectabilis* chloroplast genome.

ID	Repeat Motif	Length(bp)	Start	End	Region	Gene	ID	Repeat Motif	Length(bp)	Start	End	Region	Gene
1	(A)8	8	131	138	LSC		137	(TTC)3	9	69005	69013	LSC	
2	(TA)7	14	264	277	LSC		138	(TAT)3	9	69354	69362	LSC	
3	(ATA)3	9	431	439	LSC		139	(AAC)3	9	69652	69660	LSC	*rps18*
4	(T)10	10	457	466	LSC		140	(T)8	8	70368	70375	LSC	*rpl20*
5	(A)9	9	1721	1729	LSC		141	(T)13	13	70579	70591	LSC	
6	(T)10	10	1812	1821	LSC		142	(A)16	16	70656	70671	LSC	
7	(A)8	8	3752	3759	LSC	*trnK-UUU*	143	(T)8	8	71892	71899	LSC	*clpP*
8	(T)8	8	3901	3908	LSC	*trnK-UUU*	144	(T)8	8	72052	72059	LSC	*clpP*
9	(A)13	13	4738	4750	LSC		145	(A)9	9	72066	72074	LSC	*clpP*
10	(T)9	9	5819	5827	LSC	*rps16*	146	(A)9	9	72824	72832	LSC	*clpP*
11	(A)10	10	6567	6576	LSC		147	(T)8	8	72946	72953	LSC	*clpP*
12	(T)9	9	6650	6658	LSC		148	(T)11	11	73035	73045	LSC	*clpP*
13	(A)8	8	6679	6686	LSC		149	(T)10	10	73061	73070	LSC	*clpP*
14	(A)9	9	7367	7375	LSC		150	(T)12	12	73111	73122	LSC	*clpP*
15	(T)10	10	7395	7404	LSC		151	(A)10	10	73133	73142	LSC	*clpP*
16	(T)12	12	7406	7417	LSC		152	(A)11	11	73519	73529	LSC	
17	(A)8	8	8040	8047	LSC		153	(A)10	10	73638	73647	LSC	
18	(T)8	8	8252	8259	LSC		154	(TTG)3	9	74714	74722	LSC	*psbB*
19	(A)9	9	8433	8441	LSC		155	(TCT)3	9	75347	75355	LSC	*psbB*
20	(AT)5	10	8595	8604	LSC		156	(T)8	8	75524	75531	LSC	
21	(TAT)3	9	8736	8744	LSC		157	(T)9	9	75596	75604	LSC	

22	(T)11	11	9074	9084	LSC		158	(ATT)4	12	75972	75983	LSC	*psbN*
23	(A)9	9	9189	9197	LSC		159	(TTTC)3	12	76423	76434	LSC	
24	(T)11	11	9478	9488	LSC	*trnG-UCC*	160	(A)10	10	76640	76649	LSC	*petB*
25	(T)9	9	9610	9618	LSC	*trnG-UCC*	161	(T)8	8	76745	76752	LSC	*petB*
26	(T)11	11	9713	9723	LSC	*trnG-UCC*	162	(A)8	8	76941	76948	LSC	*petB*
27	(A)8	8	10268	10275	LSC		163	(A)11	11	76974	76984	LSC	*petB*
28	(GTCT)3	12	11483	11494	LSC	*atpA*	164	(A)10	10	76998	77007	LSC	*petB*
29	(A)10	10	12038	12047	LSC		165	(A)10	10	78115	78124	LSC	
30	(A)9	9	12733	12741	LSC	*atpF*	166	(T)9	9	78375	78383	LSC	*petD*
31	(T)8	8	12782	12789	LSC	*atpF*	167	(T)9	9	78386	78394	LSC	*petD*
32	(AAC)3	9	14911	14919	LSC	*atpI*	168	(T)10	10	79462	79471	LSC	
33	(TTA)4	12	15723	15734	LSC		169	(T)10	10	79741	79750	LSC	*rpoA*
34	(T)9	9	16807	16815	LSC	*rpoC2*	170	(TTC)3	9	79835	79843	LSC	*rpoA*
35	(T)11	11	18697	18707	LSC	*rpoC2*	171	(T)10	10	81677	81686	LSC	
36	(A)8	8	18841	18848	LSC	*rpoC2*	172	(T)11	11	81966	81976	LSC	*rps8*
37	(AT)5	10	20047	20056	LSC	*rpoC2*	173	(T)9	9	82655	82663	LSC	
38	(TTA)3	9	21112	21120	LSC	*rpoC1*	174	(T)8	8	83371	83378	LSC	*rpl16*
39	(TTC)3	9	22303	22311	LSC	*rpoC1*	175	(T)8	8	83841	83848	LSC	*rpl16*
40	(A)8	8	22487	22494	LSC	*rpoC1*	176	(T)8	8	84094	84101	LSC	*rpl16*
41	(T)11	11	22809	22819	LSC	*rpoC1*	177	(A)9	9	84206	84214	LSC	
42	(A)8	8	22965	22972	LSC	*rpoC1*	178	(ATT)3	9	84283	84291	LSC	*rps3*
43	(T)11	11	23186	23196	LSC	*rpoC1*	179	(T)9	9	85787	85795	IR	*rps19*
44	(T)8	8	26326	26333	LSC	*rpoB*	180	(T)11	11	85825	85835	IR	
45	(T)10	10	26430	26439	LSC	*rpoB*	181	(CTT)3	9	85927	85935	IR	*rpl2*

46	(A)8	8	27308	27315	LSC		182	(G)8	8	89125	89132	IR	*ycf2*
47	(TAT)3	9	27396	27404	LSC		183	(A)9	9	90264	90272	IR	*ycf2*
48	(T)10	10	27566	27575	LSC		184	(AGT)3	9	90783	90791	IR	*ycf2*
49	(T)8	8	27785	27792	LSC		185	(TCT)3	9	90920	90928	IR	*ycf2*
50	(A)8	8	27839	27846	LSC		186	(CTT)3	9	91026	91034	IR	*ycf2*
51	(T)10	10	28355	28364	LSC		187	(GGT)3	9	91290	91298	IR	*ycf2*
52	(T)8	8	29308	29315	LSC		188	(TGA)3	9	91526	91534	IR	*ycf2*
53	(CTTTA)4	20	29584	29603	LSC		189	(GAA)3	9	92786	92794	IR	*ycf2*
54	(A)10	10	29652	29661	LSC		190	(AGA)3	9	96085	96093	IR	*ndhB*
55	(T)9	9	29942	29950	LSC		191	(AGA)3	9	97512	97520	IR	*ndhB*
56	(A)16	16	30144	30159	LSC		192	(T)8	8	99873	99880	IR	
57	(T)10	10	30184	30193	LSC		193	(AAG)3	9	100423	100431	IR	
58	(A)9	9	30508	30516	LSC		194	(T)11	11	100473	100483	IR	
59	(T)10	10	30988	30997	LSC		195	(T)8	8	103343	103350	IR	*trnI-GAU*
60	(TTA)3	9	31190	31198	LSC		196	(T)8	8	104655	104662	IR	*trnA-UGC*
61	(TAA)3	9	31250	31258	LSC		197	(CTG)3	9	105137	105145	IR	*rrn23*
62	(A)8	8	31335	31342	LSC		198	(AGGT)3	12	106654	106665	IR	*rrn23*
63	(ATAAG)3	15	32098	32112	LSC		199	(CAA)3	9	110198	110206	IR	*ycf1*
64	(T)9	9	32277	32285	LSC		200	(T)8	8	110882	110889	IR	*ycf1*
65	(ATT)4	12	33055	33066	LSC		201	(A)8	8	111767	111774	SSC	*ndhF*
66	(T)9	9	33070	33078	LSC		202	(T)8	8	111884	111891	SSC	*ndhF*
67	(T)9	9	33102	33110	LSC		203	(TAA)3	9	112719	112727	SSC	*ndhF*
68	(G)10	10	34820	34829	LSC	*psbC*	204	(A)8	8	113205	113212	SSC	*ndhF*
69	(T)9	9	35796	35804	LSC		205	(A)9	9	113659	113667	SSC	

70	(ATA)3	9	36822	36830	LSC		206	(T)8	8	113685	113692	SSC	
71	(AT)5	10	36828	36837	LSC		207	(T)8	8	114344	114351	SSC	*rpl32*
72	(TCT)3	9	37660	37668	LSC	*rps14*	208	(T)9	9	114506	114514	SSC	
73	(ATG)3	9	39192	39200	LSC	*psaB*	209	(T)10	10	114699	114708	SSC	
74	(TTG)3	9	40797	40805	LSC	*psaA*	210	(T)8	8	114878	114885	SSC	
75	(GCA)3	9	41090	41098	LSC	*psaA*	211	(T)11	11	114897	114907	SSC	
76	(T)11	11	42419	42429	LSC		212	(A)8	8	115125	115132	SSC	
77	(AT)5	10	42798	42807	LSC		213	(T)8	8	115433	115440	SSC	
78	(T)8	8	42837	42844	LSC		214	(TTC)3	9	116590	116598	SSC	
79	(T)11	11	42856	42866	LSC		215	(A)8	8	116644	116651	SSC	*ndhD*
80	(TATT)3	12	42885	42896	LSC		216	(T)8	8	117229	117236	SSC	*ndhD*
81	(ATT)3	9	42901	42909	LSC		217	(TAT)3	9	117290	117298	SSC	*ndhD*
82	(A)11	11	42968	42978	LSC		218	(A)9	9	118222	118230	SSC	*ndhD*
83	(T)10	10	44022	44031	LSC	*ycf3*	219	(AAT)4	12	118866	118877	SSC	
84	(T)8	8	44692	44699	LSC	*ycf3*	220	(A)9	9	118878	118886	SSC	
85	(A)10	10	45095	45104	LSC	*ycf3*	221	(A)9	9	119410	119418	SSC	
86	(T)9	9	45398	45406	LSC		222	(TTC)3	9	121291	121299	SSC	*ndhA*
87	(GTA)3	9	45480	45488	LSC		223	(T)10	10	121717	121726	SSC	*ndhA*
88	(T)11	11	46221	46231	LSC		224	(T)8	8	121729	121736	SSC	*ndhA*
89	(TA)6	12	47115	47126	LSC		225	(T)8	8	121911	121918	SSC	*ndhA*
90	(A)9	9	47288	47296	LSC		226	(T)9	9	124413	124421	SSC	*rps15*
91	(T)11	11	47656	47666	LSC		227	(TAA)3	9	124679	124687	SSC	
92	(TA)6	12	47738	47749	LSC		228	(ATT)3	9	125159	125167	SSC	*ycf1*
93	(A)8	8	47807	47814	LSC		229	(T)12	12	125699	125710	SSC	*ycf1*

94	(A)9	9	48573	48581	LSC	*trnL-UAA*	230	(T)8	8	125864	125871	SSC	*ycf1*
95	(T)10	10	48640	48649	LSC	*trnL-UAA*	231	(T)11	11	125892	125902	SSC	*ycf1*
96	(T)9	9	50548	50556	LSC		232	(T)10	10	126081	126090	SSC	*ycf1*
97	(AAC)3	9	51205	51213	LSC	*ndhK*	233	(TTA)4	12	126138	126149	SSC	*ycf1*
98	(T)9	9	51354	51362	LSC		234	(T)9	9	126417	126425	SSC	*ycf1*
99	(TAT)3	9	52183	52191	LSC		235	(T)9	9	126510	126518	SSC	*ycf1*
100	(A)8	8	52598	52605	LSC		236	(TAAT)3	12	126585	126596	SSC	*ycf1*
101	(T)8	8	52781	52788	LSC	*trnV-UAC*	237	(T)14	14	126626	126639	SSC	*ycf1*
102	(T)10	10	53706	53715	LSC		238	(T)8	8	127357	127364	SSC	*ycf1*
103	(T)10	10	55700	55709	LSC	*atpB*	239	(T)17	17	127555	127571	SSC	*ycf1*
104	(ATA)4	12	55754	55765	LSC	*atpB*	240	(ATTT)3	12	127594	127605	SSC	*ycf1*
105	(A)8	8	55994	56001	LSC		241	(T)10	10	127693	127702	SSC	*ycf1*
106	(T)8	8	58491	58498	LSC		242	(T)13	13	127803	127815	SSC	*ycf1*
107	(AGT)3	9	59205	59213	LSC	*accD*	243	(AAT)3	9	128025	128033	SSC	*ycf1*
108	(GGA)3	9	59567	59575	LSC	*accD*	244	(T)8	8	128161	128168	SSC	*ycf1*
109	(A)9	9	60246	60254	LSC		245	(T)9	9	128210	128218	SSC	*ycf1*
110	(T)10	10	60519	60528	LSC		246	(A)9	9	128326	128334	SSC	*ycf1*
111	(A)10	10	60556	60565	LSC		247	(T)8	8	128690	128697	SSC	*ycf1*
112	(AAT)4	12	60581	60592	LSC		248	(T)9	9	128973	128981	SSC	*ycf1*
113	(T)8	8	60641	60648	LSC		249	(T)9	9	129103	129111	SSC	*ycf1*
114	(TAAT)3	12	60710	60721	LSC		250	(A)8	8	129349	129356	IR	*ycf1*
115	(AAAT)3	12	62003	62014	LSC		251	(TTG)3	9	130032	130040	IR	*ycf1*
116	(T)10	10	62449	62458	LSC		252	(CTAC)3	12	133571	133582	IR	*rrn23*
117	(A)9	9	62542	62550	LSC		253	(CAG)3	9	135093	135101	IR	*rrn23*

118	(A)11	11	62666	62676	LSC	*cemA*	254	(A)8	8	135576	135583	IR	*trnA-UGC*
119	(AT)5	10	63710	63719	LSC	*petA*	255	(A)8	8	136888	136895	IR	*trnI-GAU*
120	(CAG)3	9	64266	64274	LSC	*petA*	256	(A)11	11	139755	139765	IR	
121	(A)8	8	64639	64646	LSC		257	(CTT)3	9	139807	139815	IR	
122	(T)14	14	64705	64718	LSC		258	(A)8	8	140358	140365	IR	
123	(A)11	11	65230	65240	LSC		259	(TTC)3	9	142717	142725	IR	*ndhB*
124	(TAA)3	9	65490	65498	LSC		260	(TCT)3	9	144145	144153	IR	*ndhB*
125	(TTTC)3	12	65853	65864	LSC		261	(TTC)3	9	147444	147452	IR	*ycf2*
126	(A)10	10	66047	66056	LSC	*psbF*	262	(TCA)3	9	148704	148712	IR	*ycf2*
127	(A)8	8	66767	66774	LSC		263	(ACC)3	9	148940	148948	IR	*ycf2*
128	(T)8	8	67465	67472	LSC		264	(AAG)3	9	149204	149212	IR	*ycf2*
129	(AGG)3	9	67650	67658	LSC		265	(AGA)3	9	149310	149318	IR	*ycf2*
130	(A)8	8	67898	67905	LSC		266	(ACT)3	9	149447	149455	IR	*ycf2*
131	(A)10	10	68202	68211	LSC		267	(T)9	9	149966	149974	IR	*ycf2*
132	(A)9	9	68260	68268	LSC		268	(C)8	8	151106	151113	IR	*ycf2*
133	(T)8	8	68557	68564	LSC	*psaJ*	269	(GAA)3	9	154302	154310	IR	*rpl2*
134	(A)8	8	68671	68678	LSC		270	(A)11	11	154403	154413	IR	
135	(A)8	8	68773	68780	LSC		271	(A)9	9	154443	154451	IR	
136	(TAT)3	9	68920	68928	LSC								
